# MiMeJF: Application of Coupled Matrix and Tensor Factorization (CMTF) for Enhanced Microbiome-Metabolome Multi-Omic Analysis

**DOI:** 10.3390/metabo15010051

**Published:** 2025-01-14

**Authors:** Zheyuan Ou, Xi Fu, Dan Norbäck, Ruqin Lin, Jikai Wen, Yu Sun

**Affiliations:** 1 Guangdong Provincial Key Laboratory of Protein Function and Regulation in Agricultural Organisms, College of Life Sciences, South China Agricultural University, Guangzhou 510642, China; the_o.scau.edu.cn@stu.scau.edu.cn (Z.O.); linruqin@scau.edu.cn (R.L.); jkwen@scau.edu.cn (J.W.); 2 Guangdong Provincial Engineering Research Center of Public Health Detection and Assessment, School of Public Health, Guangdong Pharmaceutical University, Guangzhou 510006, China; fuxi@gdpu.edu.cn; 3 Occupational and Environmental Medicine, Department of Medical Science, University Hospital, Uppsala University, 75237 Uppsala, Sweden; dan.norback@medsci.uu.se

**Keywords:** biomarker identification, latent factor, dimension reduction, functional pathway analysis, multi-omics analysis

## Abstract

**Background/Objectives**: The integration of microbiome and metabolome data could unveil profound insights into biological processes. However, widely used multi-omic data analyses often employ a stepwise mining approach, failing to harness the full potential of multi-omic datasets and leading to reduced detection accuracy. Synergistic analysis incorporating microbiome/metabolome data are essential for deeper understanding. **Method**: This study introduces a Coupled Matrix and Tensor Factorization (CMTF) framework for the joint analysis of microbiome and metabolome data, overcoming these limitations. Two CMTF frameworks were developed to factorize microbial taxa, functional pathways, and metabolites into latent factors, facilitating dimension reduction and biomarker identification. Validation was conducted using three diverse microbiome/metabolome datasets, including built environments and human gut samples from inflammatory bowel disease (IBD) and COVID-19 studies. **Results**: Our results revealed biologically meaningful biomarkers, such as *Bacteroides vulgatus* and acylcarnitines associated with IBD and pyroglutamic acid and p-cresol associated with COVID-19 outcomes, which provide new avenues for research. The CMTF framework consistently outperformed traditional methods in both dimension reduction and biomarker detection, offering a robust tool for uncovering biologically relevant insights. **Conclusions**: Despite its stringent data requirements, including the reliance on stratified microbial-based pathway abundances and taxa-level contributions, this approach provides a significant step forward in multi-omics integration and analysis, with potential applications across biomedical, environmental, and agricultural research.

## 1. Introduction

The confluence of microbiome and metabolome data has contributed significantly to a comprehensive biological understanding. By integration of these datasets, many studies can provide a holistic view, allowing researchers to delve into the intricate interplay between microbial communities, their metabolic by-products, and their implications in human diseases, including rhinitis, asthma, inflammatory bowel disease (IBD), and non-alcoholic fatty liver disease [1,2,3,4,5,6,7]. Such studies are pivotal in deciphering complex host-microbe dynamics, disease mechanisms, and therapeutic potentials. Therefore, the integration of metabolomic data into microbial community-related research has become an increasingly important trend.

However, there are also major challenges in multi-omics data analysis of microbiome-metabolome research. First, the biomarker identification accuracy is low; many true biomarkers are undetected, while false positive results are reported. False positives are driven by uncorrected statistical testing, indirect associations, and batch effects, leading to spurious microbe-metabolite correlations. For example, methods such as Pearson or Spearman correlation have high false-positive rates, as they may detect non-causal associations or confounding relationships [8]. Experimental studies have shown that some of these correlations are artifacts with no underlying biological mechanism to validate them. [9,10]. Another limitation arises from the segmented single-omics analysis approach, where researchers identify candidate features from microbiome or metabolome datasets independently before integrating them in multi-omics analyses. This stepwise process often introduces irrelevant or noisy features (e.g., metabolites unrelated to microbial activity), which inflate the risk of false-positive associations during downstream analysis. For example, many researchers utilize tools like LEfSe [11] to first identify microbial or metabolite biomarkers from single-omics. Only after this initial analysis do they combine the results from both data [12,13,14,15,16]. By analyzing datasets in isolation, researchers miss critical interdependencies and synergies that exist between microbiome and metabolome data, reducing the power to detect true biological relationships. Besides the two primary challenges, various bioinformatics programs exhibit other limitations, including the absence of consideration of microbial functional profile and dimension reduction/sample clustering tools [17,18,19,20].

Matrix Factorization, Tensor Component Analysis, and Coupled Matrix and Tensor Factorization (CMTF) are powerful analytical tools initially developed for fields such as signal processing, data mining, network analysis, and machine learning [21,22,23]. Unlike traditional methods that analyze one dataset at a time, CMTF allows the simultaneous decomposition of multiple interconnected datasets into shared patterns, revealing insights that may be obscured in stepwise approaches. For example, while traditional methods may identify associations separately in microbiome and metabolome data, CMTF identifies interrelations and shared drivers across datasets, enhancing sensitivity and recall in identifying biologically meaningful features while also minimizing the inclusion of irrelevant features. These unique capabilities make CMTF particularly well-suited for addressing the challenges of multi-omics data integration.

In this study, we employed the CMTF framework to analyze multi-omics datasets, integrating microbial taxa, functional pathways, and metabolites into a unified analytical process. By capturing the relationships between these components, CMTF enables accurate dimension reduction and biomarker identification, minimizing the occurrence of false positives. Validation using datasets from built environments and human gut studies (e.g., IBD and COVID-19) demonstrated that CMTF consistently outperformed conventional methods, such as LEfSe, in both dimension reduction and biomarker detection. Notably, CMTF identified biologically meaningful biomarkers, including *Bacteroides vulgatus* and acylcarnitines associated with IBD, as well as pyroglutamic acid and p-cresol associated with COVID-19 outcomes. These findings highlight the potential of CMTF as a robust tool for uncovering biologically relevant insights and addressing key limitations in microbiome-metabolome research.

## 2. Materials and Methods

### 2.1. Basic Coupled Matrix-Tensor Factorization (CMTF) Framework

The proposed program “MiMeJF” (Microbial functional profile-Metabolome Joint Factorization) is built upon Coupled Matrix-Tensor Factorization [24,25], a type of latent factor model. In this model, heterogeneous data from different sources are structured into a coupled matrix-tensor with one shared dimension. We first built a basic CMTF framework, which assumes that the tensor and matrix always share all latent factors [26]. We obtained stratified microbiome functional data through shotgun metagenomics and HUMAnN3 annotation [27] and metabolomic data through untargeted liquid-chromatogram mass spectrum (LC-MS), respectively. The stratified functional data is pathway abundance at species per sample in the form of a matrix; thus, they can be stacked into a tensor T ∈ $R^{I_{1}\times I_{2}\times I_{3}}$, where $I_{1}$, $I_{2}$, $I_{3}$ represent functional pathways, microbes, and samples, respectively. Similarly, the metabolomic data can be structured as a matrix M $\in R^{I_{3}\times V}$ to represent metabolite intensity in each sample, where $I_{3}$ and V represent samples and metabolites, respectively. In our approach, the microbiome data used is microbial functional annotation abundance data, not merely taxa abundance data. This enables us to organize the data in a pathway × microbe × sample format—naturally suited for tensor representation. In contrast, the metabolomic data in our study does not include pathway-level annotations, thereby limiting it to a matrix (metabolites × samples) structure. Let T and M share the $I_{3}$ mode, thus they can be factorized as follows:

$$M\approx \sum_{r=1}^{R}a_{r}⊗d_{r}=\hat{M},\;T\approx \sum_{r=1}^{R}a_{r}⊗b_{r}⊗c_{r}=\hat{T}$$

where ⊗ denotes outer products, a represents the factor matrix of the shared sample’s mode, and b, c, d represent factor matrices of microbe, functional pathway, and metabolite, respectively. *R* represents the number of latent factors.

Both the tensor and matrix are formulated as the sum of the outer products of factor matrices. After structuring two omics data into the CMTF framework, we implemented an alternating least squares (ALS) algorithm for optimization. The least squares estimation for each factor matrix is formulated as follows:

$$a=\left[T_{\left(1\right)}:M\right]{\left[{\left(c⊙b\right)}^{T}:d^{T}\right]}^{T}{\left[b^{T}b*c^{T}c\right]}^{†}$$

$$b=T_{\left(2\right)}[c⊙a]{\left[a^{T}a*c^{T}c\right]}^{†}$$

$$c=T_{\left(3\right)}[b⊙a]{\left[a^{T}a*b^{T}b\right]}^{†}$$

$$d=M^{T}{\left(a^{†}\right)}^{T}$$

where denotes the horizontal concatenation of matrices, ⊙ denotes the khatri-rao product, ∗ denotes the Hadamard product, † denotes the generalized inverse of matrices and $T_{\left(n\right)}$ represents the *n*th matricization of tensor.

Data can be reconstructed by the sum of the outer products of factor matrices. Thus, the objective function is the root mean squared error (RMSE) between the reconstructed and original data. The optimization procedure stops when the relative change of RMSE is less than $1\times 10^{-6}$, expressed as follows:

$$\frac{\left|{rmse}_{current}-{rmse}_{previous}\right|}{{rmse}_{previous}}\;\leq 1\times 10^{-6}$$

### 2.2. Advanced CMTF Framework

Besides the basic framework, MiMeJF also implements an advanced CMTF framework. In the advanced framework, tensor and matrix are not assumed to share all the latent factors. Instead, both the shared and unshared components are modeled independently [26]. Certain latent factors jointly explain variability across both datasets (shared), while others are specific to either the microbiome or the metabolome (unshared). This design reflects the fact that some microbial processes directly influence metabolite profiles, whereas other processes are independent of metabolite production. The factorization can be expressed as follows:

$$M\approx \sum_{r=1}^{R}\sigma_{r}⊗a_{r}⊗d_{r}=\hat{M},T\approx \sum_{r=1}^{R}\lambda_{r}⊗a_{r}⊗b_{r}⊗c_{r}=\hat{T}$$

where σ and λ represent weights of latent factors in tensor and matrix, respectively. Since distinct weights have been introduced into the model, the objective function can be expressed as follows:

$$f={\left||T-\hat{T}|\right|}^{2}+{\left||M-\hat{M}|\right|}^{2}+\beta {\left||\lambda |\right|}^{1}+\beta {\left||\sigma |\right|}^{1}+\alpha \sum_{r=1}^{R}{\left(\left|\left|a\right|\right|-1\right)}^{2}+\alpha \sum_{r=1}^{R}{\left(\left|\left|b\right|\right|-1\right)}^{2}+\alpha \sum_{r=1}^{R}{\left(\left|\left|c\right|\right|-1\right)}^{2}\;+\alpha \sum_{r=1}^{R}{\left(|\left|v\right||-1\right)}^{2}\;\;\;\;\;\;\;\;\;\;\;\;\;\;\;\;\;\;$$

where β represents the penalty parameter of weights and α represents the penalty parameters of columns of factor matrices.

In the advanced framework, we replaced ALS with a gradient-based optimization to enhance computational accuracy. The gradient expression for the variables to be solved is expressed as follows:

∂a=T^(1)−T1λT⊙c⊙b+M^−Mvσ+α(a−a¯)∂b=T^(2)−T2λT⊙c⊙a+α(b−b¯)∂c=T^(3)−T3λT⊙b⊙a+α(c−c¯)∂v=(M^−M)Taσ+α(v−v¯)∂λr=T^−T×1ar×2br×3cr+β2 λrλr2−ϵ∂σr=arTM^−Mvr+β2 λrλr2−ϵ

where ϵ represents a sufficiently small variable introduced for derivation, and $\times_{n}$ denotes the tensor-vector product in the *n*th mode. $\bar{a}$ represents the arithmetic mean of a vector a.

We used the BFGS (Broyden–Fletcher–Goldfarb–Shanno) method implemented in SciPy (v1.5.4) as the default solver to perform gradient-based optimization, while other solvers available in scipy.optimize.minimize can also be used. Gradient-based optimization treats every data entry as a variable in each iteration. Therefore, the computational cost can be significantly higher than with the ALS in the basic CMTF framework, especially when dealing with large datasets, a common scenario in microbiome/metabolome datasets.

### 2.3. Omics Data Preprocessing

Before structuring them into the CMTF framework, both the microbial functional profile and metabolome data require preprocessing. We first performed functional annotation on microbial taxa abundance data using HUMAn3 with MetaCyc as the reference database. After obtaining the stratified functional profile from HUMAnN3, we used the “logstack scaling” method, as described in the HUMAnN3 visualization function [27], to scale the abundance data. This process involved a log transformation of the total community abundance, followed by a linear scaling of species contributions within this log-transformed total abundance. For the metabolome data, several researchers have treated metabolome data as compositional data and achieved decent results [28,29], so we performed a centered log-ratio transformation for each sample.

### 2.4. Cross-Validation

Cross-validation is a widely used technique to prevent overfitting and optimize model parameters; it can be used for finding the optimal number of latent factors. Since CMTF is an unsupervised model, we randomly split the tensor and matrix into training and test sets by masked tensors/matrices, where excluded entities are masked by 0. After factorization of the training set, the factor matrices were used to reconstruct data. This enabled the computation of errors for both the training and test sets as follows:

$$train\;rmse=W_{t}\frac{{\left||\omega_{t}*(T-\hat{T})|\right|}_{2}}{{\left||\omega_{t}*T|\right|}_{2}}+W_{m}\frac{{\left||\omega_{m}*(M-\hat{M})|\right|}_{2}}{{\left||\omega_{m}*M|\right|}_{2}}$$

$$test\;rmse=W_{t}\frac{{\left||\left(1-\omega_{t}\right)*(T-\hat{T})|\right|}_{2}}{{\left||(1-\omega_{t})*T|\right|}_{2}}+W_{m}\frac{{\left||\left(1-\omega_{m}\right)*(M-\hat{M})|\right|}_{2}}{{\left||(1-\omega_{m})*M|\right|}_{2}}$$

where ω represents mask tensor/matrix, and W represents weights for tensor/matrix, which is decided by the ratio of the data size of the tensor/matrix to the total size.

### 2.5. Quantification of Group Separation and Dimension Reduction

A primary goal of MiMeJF is dimension reduction. After factorization, the loadings of samples in different latent factors can be observed. Since MiMeJF is built upon an unsupervised model, correctly interpreting these latent factors is crucial. One can attribute practical meaning to latent factors by identifying the specific distribution of the sample loading values. However, it might remain ambiguous without proper quantification. Therefore, we implemented a universal quantification approach based on the Mahalanobis distance [30] to quantify whether samples from different groups separated significantly.

Since Mahalanobis distance only performs well in two-dimensional space, we first projected samples onto the two latent factors contributing the most variance. The squared Mahalanobis distance between groups in this 2D space was then computed as follows:

$$D^{2}={\left(\bar{y}-\bar{x}\right)}^{T}{Cov}^{-1}(\bar{y}-\bar{x})$$

After computing the distance, we constructed a variable which could be expressed as follows:

$$f=\frac{s_{1}+s_{2}-n-1}{n\left(s_{1}+s_{2}-2\right)}\times \frac{s_{1}s_{2}}{s_{1}+s_{2}}\times D^{2}$$

where $s_{1}$ and $s_{2}$ are the sample size of each group, and n is the number of involved latent factors. Consistent with this 2D representation, we set *n* = f when calculating the Mahalanobis distance, ensuring alignment between data visualization and distance computation.

This variable is an F-distribution, represented as $F(n,s_{1}+s_{2}-n-1)$. A hypothesis test can be conducted to determine whether the group separation is significantly significant.

### 2.6. Evaluation of Biomarker Selection

MiMeJF projects original omics data features onto latent factors to disentangle the relationships among these features. Each latent factor represents a specific data variation, and microbes/metabolites with high absolute loading values on these latent factors are considered characteristic biomarkers. Since a large number of false positive biomarkers may be produced due to the complexity of omics data [31,32], our main concern in biomarker identification is the true positive rates. We evaluated top k biological features from the latent factor matrices to determine whether they were truly associated with specific phenotypes or any other biological groupings. The top k hit ratio can be computed as follows:

$$top\;k\;hit\;ratio=\frac{TruePositive\;biomarkers\;in\;top\;k\;biomarkers\;}{top\;k\;biomarkers}$$

By plotting a top k hit ratio curve, we benchmarked and compared different biomarker selection approaches.

The script has been stored in the GitHub repo: https://github.com/NRX-044/MiMeJF (accessed on 13 January 2025). Also, we have uploaded all relevant materials, including the analysis notebook, figures, and tables in the study, to Zenodo at the following link: https://zenodo.org/records/14279380 (accessed on 13 January 2025).

## 3. Results

### 3.1. Program Design and Implementation

The core of MiMeJF is the factorization framework. Two computational frameworks are implemented in MiMeJF: a basic framework that ensures accuracy and rapid performance and an advanced framework that can further reveal weights and contributions from microbiome and metabolome data to latent factors but at a significantly higher computational cost. Both are designed to jointly process stratified microbial functional profiles and metabolome data.

Besides the factorization model, MiMeJF provides straightforward visualization and evaluation functions. Analogous to PCA, Sample loadings on latent factors can be visualized by 2D or 3D score plots. Biomarkers, either of specific interest or with high loading values, can be presented by a loading plot. The factor matrices, as well as weights of latent factors, are stored in an object and can be output to text files. Additionally, MiMeJF provides functions to evaluate different metrics, including the root mean squared error of factorization, the significance of biological group separation, and the errors for both training and test sets in cross-validation.

MiMeJF is implemented in Python3 (v3.6.2), with the core computational part, including basic linear algebra computation and optimization algorithm, developed using NumPy (1.19.5) [33]. It can be imported as a Python module, and API support is provided.

### 3.2. Test Datasets and Sources

In this study, we utilized three multi-omics datasets to evaluate the performance of our model:

1. Aircraft Cabin Dust Microbiome and Metabolome Dataset: This dataset is from a study on dust microbiome and metabolome in aircraft cabins. It comprises 27 dust samples collected from various aircraft cabins across different airline companies [34,35];
2. Inflammatory Bowel Disease Multi-omics Dataset: This dataset is a subset of a large-scale study on inflammatory bowel diseases. The study collected samples from 132 subjects from five different hospitals, generating multi-omics data including metagenome, metatranscriptome, proteome, metabolome, etc. [36]. For our purposes, we focused on the metagenome and metabolome data;
3. Gut Microbiome and Metabolome Data of COVID-19 Patients Dataset: This dataset is generated from a study examining the gut microbiome and metabolome of 22 COVID-19 convalescents, 15 asymptomatic patients, and 12 healthy controls [37].

All data mentioned above were profiled by shotgun metagenomic sequencing and untargeted LC-MS metabolome analysis. Because the number of microbiome and metabolomic samples per subject can differ (e.g., in the IBDMDB dataset), we calculated the mean of all samples belonging to the same subject within each dataset and coupled the datasets using the subject identifier. This approach ensures that both modalities are aligned at the subject level, mitigating the impact of unequal sample sizes. Detailed dataset characteristics, including the number of samples, detected microbial taxa, annotated pathways, metabolites, and sparsity metrics, are provided in Table S1. The original contributions presented in the study are included in the article/Supplementary Material; further inquiries can be directed to the corresponding author.

**Figure 1:**
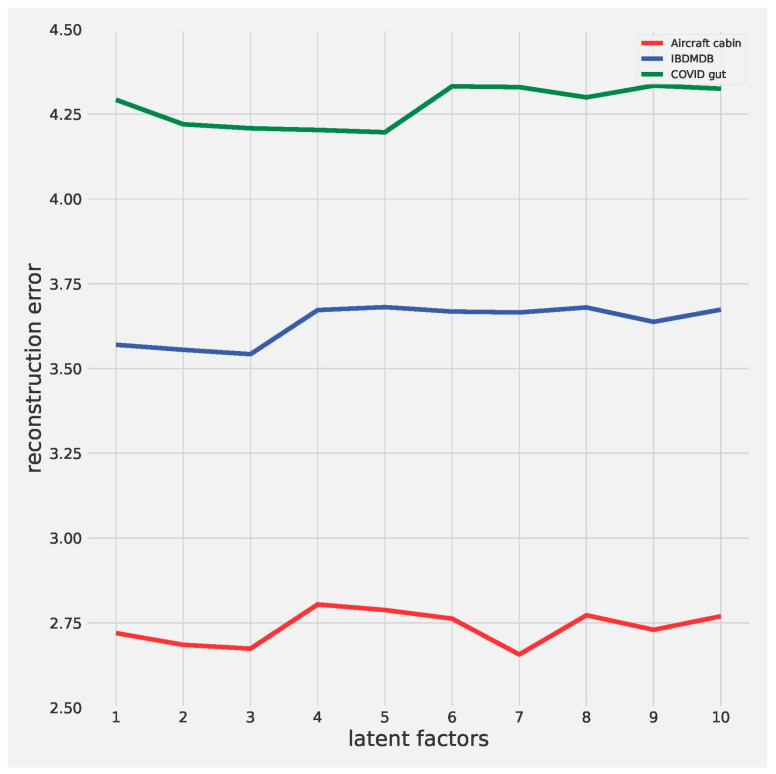
Basic CMTF performance using different numbers of latent factors. Aircraft cabin: Aircraft Cabin Dust Microbiome and Metabolome Dataset; IBDMDB: Inflammatory Bowel Disease Multi-omics Dataset; COVID gut: Gut Microbiome and Metabolome Data of COVID-19 Patients Dataset.

To investigate the impact of different numbers of latent factors on the factorization results, we applied the basic CMTF framework to the three datasets mentioned above using a range of number of latent factors from 1 to 10 (Figure 1). The results showed that all three datasets achieved sufficiently good factorization results with low RMSE when the number of latent factors was set to 3. Considering the balance between model complexity and performance, we set the number of latent factors to three for subsequent analysis on three datasets. The slight variability in RMSE across datasets may stem from differences in data complexity, encompassing factors such as dimensionality, heterogeneity of biological signals, and overall variability. Datasets with higher dimensionality or more diverse profiles can pose greater challenges for factorization, potentially yielding higher reconstruction errors.

### 3.3. Cross-Validation Using Simulated Data

To evaluate whether the basic CMTF model will generate artifact latent factors due to overfitting, we generated simulated coupled matrix-tensor data where tensor T $\in R^{a\times b\times c}$ and matrix M $\in R^{a\times d}$. We set a = 40, b = 50, c = 30, d = 20, where a, b, c, and d represent pathways, microbes, metabolites, and samples, respectively. All data entries were randomly generated from a normal distribution N(1,1). Twenty percent of the data was randomly reserved by applying a mask tensor and matrix for validation.

**Figure 2:**
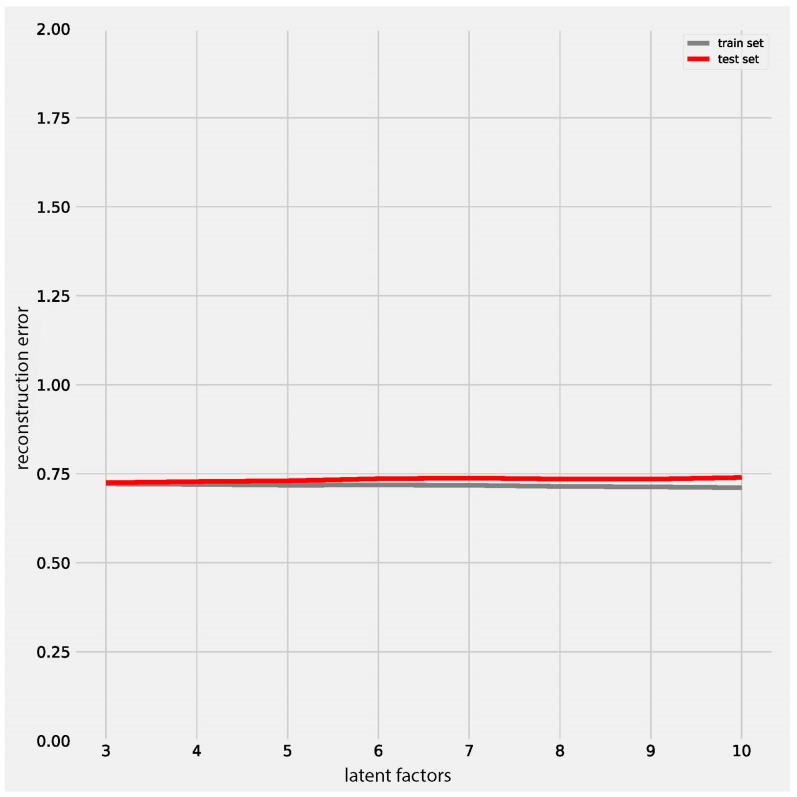
Cross-validation of CMTF on a simulated dataset.

The test set error slowly increased with the increasing number of latent factors (Figure 2), indicating that adding more latent factors might slightly increase the risk of overfitting. However, considering the minimal difference between the training and test set errors, the CMTF model remains robust with high missing values and is unlikely to overfit. Users can also utilize the cross-validation function to further find the optimal number of latent factors for their data.

### 3.4. Dimension Reduction on Aircraft Multi-Omics Dataset

Compared to analyzing single omic data, jointly analyzing microbiome and metabolome data may offer a more comprehensive understanding of the overall variations within subjects. To test this hypothesis, we evaluated group separation after dimension reduction using PCoA, a classic dimension reduction approach [38], and CMTF on aircraft microbiome and metabolome datasets. The dataset comprised 27 samples, evenly distributed among cabin samples from two airlines (A1C, A2C) and flight deck samples from one airline (A2F). The number of latent factors in CMTF was set to 3, but only the top 2 latent factors (explaining the highest variance) were used for evaluation.

**Figure 3:**
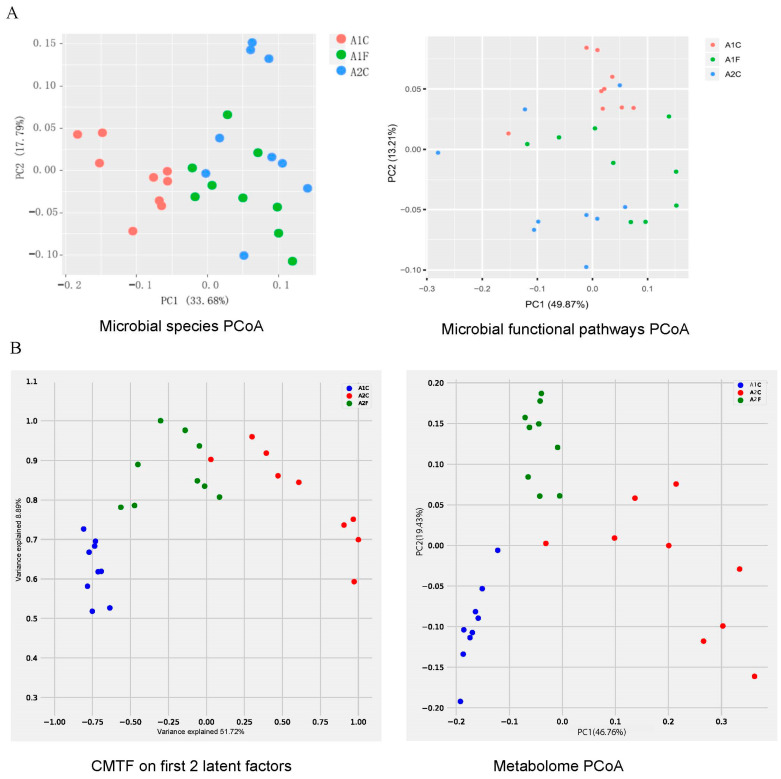
Dimension reduction performance comparison on aircraft database. (**A**) dimension reduction of microbiome data at species and pathway level. (**B**) Dimension reduction comparison between CMTF and metabolome-based PCoA.

The PCoA dimension reduction results showed that microbiome data at the species and functional pathway level discriminated between the three groups (Figure 3; *p* = 0.002 and 0.005, F-test). Extending PCoA to cabin metabolome data also revealed significant differentiation (*p* = 1.03 × 10^−7^; Figure 1). When comparing this result to the factorization result of CMTF, we observed even clearer group separation and a more significant *p*-value (Figure 3; *p* = 8.38 × 10^−13^). In this context, CMTF achieved a higher degree of separation than PCoA, indicating that integrating microbiome and metabolome data analyses may have a better chance of identifying the primary drivers of variation in the microbiome and metabolome dataset.

### 3.5. Dimension Reduction and Biomarker Identification on IBDMDB Dataset

CMTF splits data variation into multiple latent factors, enabling users to correlate features in the original data with these latent factors. This enables the identification of functional pathways strongly associated with specific metabolites and the corresponding contributing microorganisms. In the IBDMDB study, the authors identified microorganisms/metabolites relevant to IBD and dysbiosis (Supplement Tables S2 and S3) [36]. Here, we applied the CMTF model to microbiome and metabolome data to replicate the results from the original study. Since the number of samples in the microbiome data is higher than in metabolome data, directly coupling them was not feasible. To overcome this, we calculated the mean values of all samples belonging to the same subjects, and the mean microbiome data and metabolome data can couple in the ‘subjects’ dimension.

**Figure 4:**
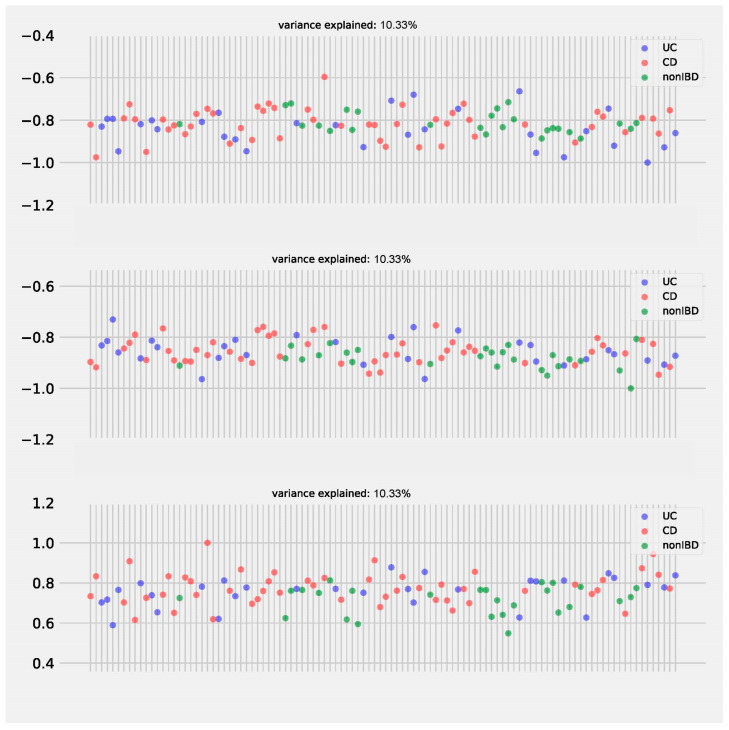
Samples loadings on 3 latent factors of CMTF on the IBDMDB dataset. The *x*-axis represents samples.

We factorized the microbiome/metabolome datasets into three latent factors. Each of these latent factors explained a comparable amount of variance (Figure 4). Using the top two latent factors, we further visualized and quantified the group separation between IBD and non-IBD subjects. Notably, while the UC (ulcerative colitis) and CD (Crohn’s disease) groups did not show significant separation (*p* = 0.152, F-test), both the UC vs. non-IBD (*p* = 0.002, F-test) and CD vs. non-IBD (*p* = 0.008, F-test) showed significant separation. In contrast, the original paper’s PCoA and PERMANOVA analysis failed to demonstrate a statistical difference between UC/CD and non-IBD groups (*p* > 0.05). Therefore, CMTF provided a more pronounced separation than PCoA, suggesting that it is more effective at capturing key microbial and metabolomic drivers that differentiate IBD from non-IBD subjects.

**Figure 5:**
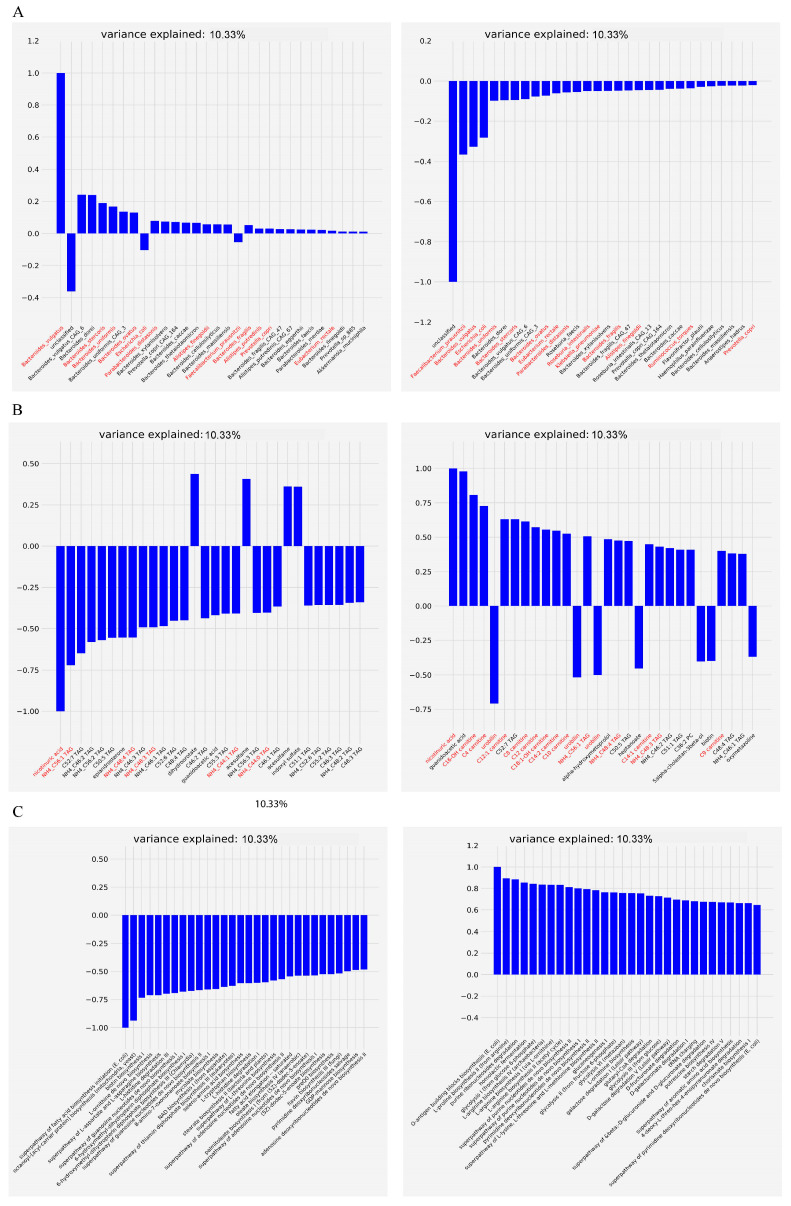
Biomarker identification (features loadings) on the first 2 latent factors of CMTF on the IBDMDB dataset. Negative loadings represent a negative association with latent factors. (**A**) Microbe loadings (**B**), metabolite loadings, and (**C**) functional pathway loadings. Texts in red font indicate validated biomarkers.

Microbes, functional pathways, and metabolites with high absolute loading values on top latent factors can be identified as biomarkers potentially associated with IBD. We selected the top 30 biological features, based on their absolute loading values, as potential biomarkers for further evaluation (Figure 5). Biomarkers with high absolute loadings on multiple latent factors suggest potential diverse interaction with other omics features, highlighting their multifaceted roles in biological processes. For example, a microbe can produce or be affected by multiple metabolites, causing different effects. The IBD-associated biomarkers, which have already been reported in IBDMDB and other studies, were highlighted in red [19,36].

CMTF successfully identified dysbiosis-associated microorganisms reported in the previous studies (Supplement Table S2; Figure 5A). For instance, the genus *Bacteroides* was dominant in microbe factor matrices, accounting for 17 out of the top 30 microbes in the first latent factors. *Bacteroides vulgatus* and *Bacteroides uniformis* had a high loading value in the first latent factor, and both of them were identified as IBD-associated microbes in the IBDMDB study. Other species, like *Prevotella copri* and *Feacalibacterium prausnitzii*, were identified in all three latent factors, which were also reported to be associated with the microbial community shift in non-IBD and IBD subjects. Besides the confirmation of previously reported species, CMTF also identified novel potential species linked with IBD. For example, *Parabacteroides distasonis*, a microorganism, has not been identified as a potential IBD-biomarker in the previous multi-omic studies [19,36], was found to be highly associated with *NKX2-3*, a known IBD-related locus [39]. The results indicate that CMTF outperforms traditional bioinformatics approaches in biomarker identification.

Similarly, CMTF also highlighted dysbiosis-associated metabolites previously reported (Supplement Table S3; Figure 5B). For instance, acylcarnitines were significantly enriched in the dysbiotic gut microbiome. While L-carnitines exhibit anti-inflammatory effects, some fatty acid-related carnitines have inflammatory effects [40]. Other key metabolites, like nicotinuric acid and urobilin, were identified as characteristic metabolites in the IBD group. CMTF also identified potential new IBD-related metabolites, like caproate and biotin, which were not identified in the IBDMDB study but reported and validated in other studies [5,41]

By examining the factor matrices of functional pathways, we observed biological connections between microbes and metabolites (Figure 5C). For example, the superpathway of fatty acid synthesis and octanoyl-[acyl-carrier protein] biosynthesis were the top two pathways in the first latent factor. Given that carnitine predominated in all three latent factors, there is a clear connection between the featured metabolites and microbial functional pathways. This could further confirm that these featured metabolites are produced by gut microbes and are truly associated with IBD.

**Figure 6:**
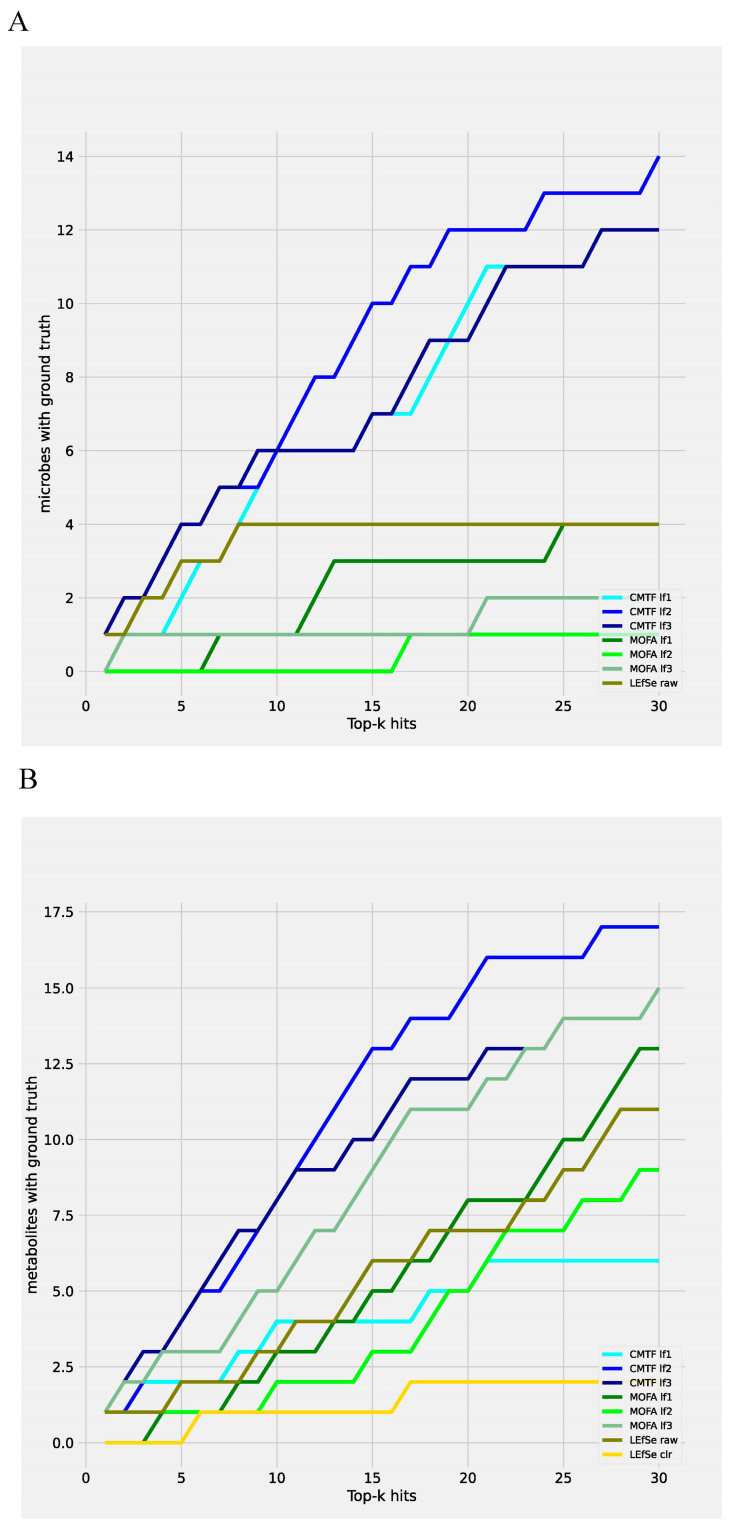
True positive biomarkers detection rate comparison among CMTF LEfSe and MOFA2. (**A**) microbes selection benchmark (**B**) metabolites selection benchmark. LEfSe raw: perform LEfSe on raw data. LEfSe clr: perform LEfSe on centered log-ratio transformed data. MOFA lf1–3: latent factor 1–3 of MOFA2.

We further benchmark the efficacy of CMTF against some state-of-the-art biomarker identification programs, including LEfSe [11] and MOFA2 [17,18] in detecting true positive biomarkers. For the evaluation, we selected the top ‘k’ potential biomarkers and counted the number of those validated. In all latent factors, CMTF outperformed LEfSe and MOFA2 in the efficiency of detecting validated microbes (Figure 6A). Additionally, CMTF outperformed LEfSe in the efficiency of detecting validated metabolites in all but one latent factor, while MOFA2 becomes competitive with CMTF in detecting metabolites (Figure 6B). These findings highlight CMTF’s robustness and a high true positive rate in biomarker identification.

### 3.6. Biomarker Identification on COVID-19 Database

**Figure 7:**
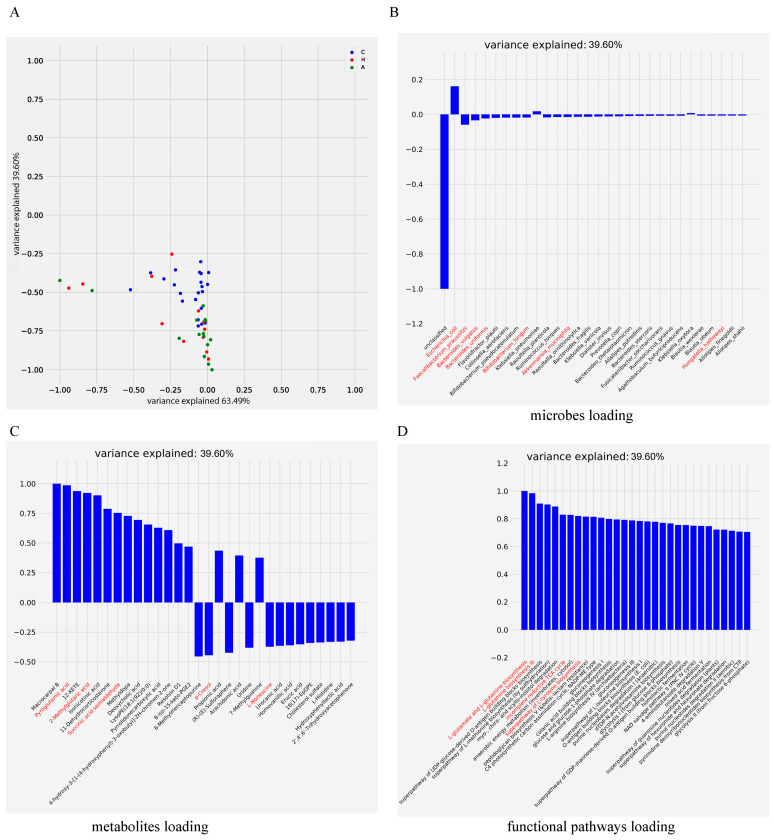
Analysis of COVID-19-related gut dataset. (**A**) Dimension reduction result of CMTF on COVID-19-related gut dataset. The figure was visualized on the first two latent factors. (**B**) Microbes, (**C**) metabolites, and (**D**) pathway loadings on the second latent factor.

Identifying true biological relevance among statistically significant results is crucial to minimizing false positive results. To further demonstrate CMTF’s potential for detecting biomarkers with putative biological relevance, we utilized a dataset that included gut microbiome and metabolome data from asymptomatic and convalescent COVID-19 cases, as well as healthy subjects [37]. The exploratory analysis in the first two latent factors showed significant separation between the asymptomatic and convalescent groups as well as the health and convalescent groups in the first two latent factors (A vs. H: *p* = 0.403, A vs. C: *p* = $7.63\times 10^{-7}$, C vs. H: *p* = 0.001; Figure 7A), consistent with previous publication. Our analysis primarily focused on the biomarkers within the second latent factor due to its clear group separation (Figure 7B–D). The CMTF model first confirmed the microbial species associated with COVID-19 in the previous study [37], including the dominant opportunistic pathogen *Escherichia coli* and the main SCFA-producing microbe, *F. prausnitzi*. *Bacteroides vulgates*, which had a high replication rate in the healthy group, were also identified.

We also identified novel microbial pathways and metabolite features previously unreported by Lin et al. We identified potential biologically relevant features: pyroglutamic acid and 2-methylglutamic acid; both require glutamate as a precursor, synthesized through glutamate and glutamine biosynthesis. Additionally, succinic acid semialdehyde is involved in the TCA cycle. p-cresol, which can be produced from tyrosine, is synthesized through the superpathway of L-tyrosine biosynthesis. These metabolites have been implicated in various studies as potentially associated with the pathogenesis of SARS-CoV-2 [42,43].

Since their true biological relevance, these co-occurrence functional pathways and metabolites may offer more confidence than evidence drawn solely from pathways or metabolites. Overall, by incorporating microbial functional pathways with metabolite datasets, CMTF may have greater chances to find real connections and biomarkers, enhancing the interpretability of the results.

### 3.7. Comparison of Basic CMTF and Advanced CMTF Model

Besides the basic CMTF model, we also implemented an advanced CMTF model designed to model variations in the tensor and matrix independently. We first examined their factorization accuracy on simulated data. The four-factor matrices used to build the simulated tensor and matrix were randomly generated from a normal distribution N(1,1). The simulated tensor and matrix had weights [1, 0.5, 1] and [1, 1, 0.5], respectively, for the three separate latent factors. This ensured that they did not share all latent factors uniformly. We performed both basic and advanced CMTF on this data for a performance comparison. The error of the advanced CMTF was significantly smaller than that of the basic CMTF (0.018 vs. 7.282; Supplement Figure S1). Further, by making the tensor and matrix weights identical, the enhanced model still showed superior factorization accuracy over the standard model (0.025 vs. 6.250; Supplement Figure S1). However, when tested on real multi-omics data, advanced CMTF did not show smaller errors over the basic model. We tested it on three previously mentioned real datasets and found that advanced CMTF had similar errors compared to basic CMTF while costing enormous computational time (Table 1). An explanation is the variance in data distribution between simulated and real datasets. In the simulated dataset, data was clean and simple, with distinct weights and normal distributions of all entries. Conversely, real multi-omics data is much more complicated. The distinction in the weights of heterogeneous data is less clear, which might compromise the optimization accuracy of advanced CMTF. Therefore, the merit of advanced CMTF in factorization accuracy warrants further validation on large-scale datasets.

**Figure 8:**
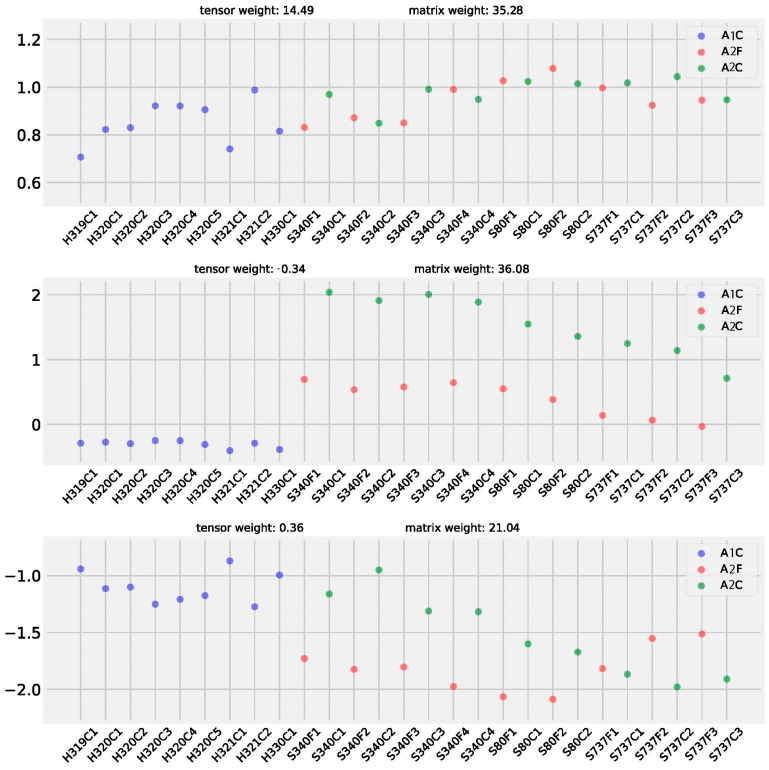
Samples loadings of advanced CMTF on flight cabin dataset, weights of microbial functional profile and metabolome profile were demonstrated respectively.

We further demonstrated the biomarker identification and variation separating capacity of the advanced CMTF model. The advanced model showed similar power in biomarker detection as the basic model (Table 2), with both models detecting very similar sets of biomarkers in the IBDMDB dataset. A strength of the advanced CMTF model is that it can show heterogeneous weights and contributions from microbiome and metabolome data to latent factors (Figure 8). In the second latent factor of the flight cabin dataset, microbiome taxa and functional data (tensor) only accounted for about 2% of the weights, while metabolome data (matrix) accounted for 39% of the weights. This suggests that the metabolome data predominantly contributed to the variation in the second latent factor, while microbiome data had a minor contribution. This result also explained the good performance of PCoA using only metabolome data (Figure 3). Overall, while the advanced CMTF has been integrated to offer an option to elucidate heterogeneous weights and contributions from multi-omics, its high computational demands, and similar biomarker identification power make the basic CMTF the more appealing choice for those only interested in biomarker identification.

## 4. Discussion

In this study, we reported a multi-omics data analysis tool for microbiome and metabolome datasets. Our findings underscored the potential pitfalls in current analytical approaches and the strength of utilizing the CMTF method to address these challenges.

There are several strengths in this study. First, our program consistently provided robust results and outperformed some widely used approaches. Incorporating additional biological information as prior data helps to filter out false positive correlations that might be mechanistically implausible [44]. By incorporating a microbial functional profile instead of a direct taxonomic profile, our approach makes use of this prior information to control the false positive rate and enhances the interpretability of the results. It provides a perspective on the connection between microbes and metabolites by linking them with functional pathways. Co-occurrence of feature microbes, pathways, and metabolites on top loadings provides a strong indication of their true biological relevance, whereas co-occurrence features without biological relevance can be considered to be excluded from further analysis, such as metabolites that are not part of pathways or are not precursors/products of pathways. Second, the advanced CMTF model offers an unsupervised way to distinguish between homogeneous variation and heterogeneous variation, addressing concerns that some popular supervised models, like OPLS and PLS, might be prone to overfitting and generating misleading artifacts [45,46].

Despite its strengths, MiMeJF has certain limitations. For instance, it requires stratified data on microbial-based pathway abundances with taxa-level contributions, which can be somewhat stringent. Additionally, the framework does not currently include metabolite pathway annotations. The program is limited to providing factorization results and basic visualizations, necessitating manual curation of co-occurring biomarkers—such as microbes, metabolites, and functional pathways—to establish their biological relevance. To enhance its utility, future versions should incorporate databases such as HMDB and MetaCyc to facilitate biological relevance searches and comparisons [47,48]. Another key limitation is the lack of a holdout dataset or independent validation set for tuning and performance evaluation. While parameters can be adjusted to optimize cross-validation statistics, relying solely on a single dataset raises the potential for overfitting. In other words, MiMeJF may fit the training data closely but perform less effectively on new, unseen data. Future research incorporating an external validation cohort or more robust cross-validation designs would help confirm the generalizability and reliability of the method.

We further compare MiMeJF with other popular multi-omics data analysis tools. For example, mixOMICS, MOFA2 has integrated several unsupervised and supervised multivariate data analysis algorithms, including PCA, CCA, PLS-DA, etc. [20]. These algorithms are capable of distinguishing samples from different groups in lower dimensions and selecting omics features simultaneously. However, most multivariate approaches in mixOMICS assume that microbiome and metabolome profiles share all latent factors with the same weights, and microbial functional profiles are not considered in either mixOMICS or MOFA2. In contrast, our advanced model in MiMeJF introduces distinct weights for microbiome and metabolome data, addressing a current shortcoming in mixOMICS. Another widely used tool, mmvec, inspired by word2vec model, offers a fundamentally different approach to multi-omics data analysis [19]. It disentangles microbe-metabolite co-occurrence probability by applying neuro network analysis on paired microbiome and metabolome data. However, its limitations are evident; mmvec cannot perform sample clustering and dimension reduction, preventing it from associating correlation results with specific phenotypes. Additionally, it can not distinguish between a negative and positive correlation. By comparison, MiMeJF not only facilitates dimension reduction but also associates biomarker clusters with specific phenotypes, paving the way for the efficient identification of pivotal biomarkers.

## Figures and Tables

**Table 1:** RMSE and computation time comparison between basic CMTF and advanced CMTF on three real datasets.

	Basic CMTF RMSE	Basic CMTF Computation Time	Advanced CMTF RMSE	Advanced CMTF Computation Time
Flight Cabin	2.67	4.04	2.80	75,641.20
IBDMDB	3.54	10.33	3.69	22,360.51
COVID-19 gut related	4.21	4.93	4.41	7909.92

**Table 2:** Validated biomarkers identified by basic CMTF and advanced CMTF on the IBDMDB dataset.

	Metabolite Markers in Top 3 Latent Factors	Microbe Markers in Top 3 Latent Factors
Advanced CMTF	Nicotinuric acid	*Faecalibacterium prausnitzii*
	NH4_C56:1 TAG	*Escherichia coli*
	NH4_C48:4 TAG	*Bacteroides uniformisv*
	NH4_C48:3 TAG	*Eubacterium rectale*
	NH4_C44:1 TAG	*Roseburia intestinalis*
	NH4_C44:0 TAG	*Ruminococcus torques*
	C16-OH carnitine	*Klebsiella pneumoniae*
	C8 carnitine	*Bacteroides stercoris*
	urobilin	*Bacteroides ovatus*
	C4 carnitine	*Bacteroides fragilis*
	C12:1 carnitine	*Prevotella copri*
	C10 carnitine	*Parabacteroides distasonis*
	C9 carnitine	*Alistipes finegoldii*
	C12 carnitine	*Alistipes putredinis*
	C14:2 carnitine	
	C14:1 carnitine	
	C18:1-OH carnitine	
Basic CMTF	Nicotinuric acid	*Bacteroides vulgatus*
	NH4_C56:1 TAG	*Bacteroides stercoris*
	NH4_C48:4 TAG	*Bacteroides uniformis*
	NH4_C48:3 TAG	*Bacteroides ovatus*
	NH4_C44:1 TAG	*Escherichia coli*
	NH4_C44:0 TAG	*Parabacteroides distasonis*
	C16-OH carnitine	*Alistipes finegoldii*
	C4 carnitine	*Faecalibacterium prausnitzii*
	urobilin	*Bacteroides fragilis*
	C12:1 carnitine	*Alistipes putredinis*
	C8 carnitine	*Prevotella copri*
	C12 carnitine	*Eubacterium rectale*
	C18:1-OH carnitine	*Roseburia intestinalis*
	C14:2 carnitine	*Klebsiella pneumoniae*
	C10 carnitine	*Ruminococcus torques*
	C14:1 carnitine	
	C9 carnitine	
	C14:2 carnitine	

## Data Availability

The original contributions presented in the study are included in the article and Supplementary Material, further inquiries can be directed to the corresponding author.

## Supplementary Materials

The following supporting information can be downloaded at https://www.mdpi.com/article/10.3390/metabo15010051/s1, Figure S1: Simulation study on the effect of different data variance and data sparisity on factorization. A: Simulated factor matrices were randomly drawn from normal distribution N(10,sigma), where sigma ranging from 1 to 10, these factor matrices were used to build simulated tensor and matrix respectively, applying identical weights on tensor and matrix. The error was evaluated on basic CMTF and advanced CMTF.B: Simulated factor matrices were randomly drawn from normal distribution N(10,1), simulated tensor and matrix were built using the same way as above. Then a proportion ranging from 0 to 0.8 of data was randomly masked from simulated tensor and matrix to simulate different data sparsity. The error was evaluated on basic CMTF and advanced CMTF; Table S1: Description of datasets used for validating the CMTF framework. The table summarizes key characteristics of each dataset, including the number of detected microbial taxa, annotated functional pathways, and metabolites, along with the total number of samples analyzed. Tensor sparsity refers to the proportion of missing or zero entries in the tensor representation of the data, while matrix sparsity refers to the proportion of missing or zero entries in the metabolomic data matrix; Table S2: IBD-related microbial biomarkers; Table S3: IBD-related metabolic biomarkers.

## Glossary

Tensor	Generalization form of data arrangement: the tensor used in this manuscript refers to a three-way data array, which is used for representing microbial pathways abundance data.
Matrix	two-way data array, which is used to represent metabolites’ abundance data.
Latent factor	Vectors capture part of data variation after factorization, and features from original data are projected onto them.
Factor matrix	A matrix containing loadings of specific features on all latent factors.
Weights of latent factors	A vector containing weights on different latent factors of one dataset, which is used in the advanced CMTF framework specifically.
CMTF	Coupled matrix-tensor factorization.
