# Supplementary material for: MiMeJF: Application of Coupled Matrix and Tensor Factorization (CMTF) for Enhanced Microbiome-Metabolome Multi-Omic Analysis
Source: Metabolites. 2025 Jan 14;15(1):51. doi: 10.3390/metabo15010051 (PMC11767930; doi:10.3390/metabo15010051)
Supplement: Supplementary file 1 [file metabolites-15-00051-s001.zip › metabolites-3309804-supplementary.pdf]

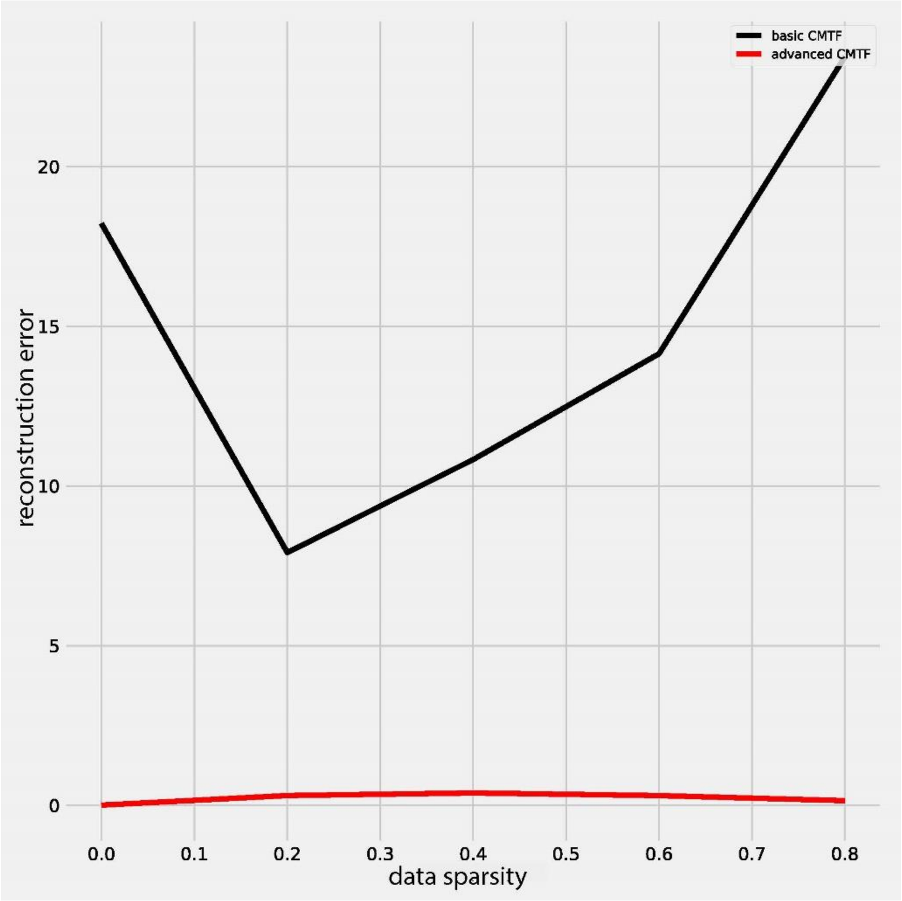

basic CMTF
advanced CMTF
20
15
10
5
0
reconstruction error
0.0
0.1
0.2
0.3
0.4
0.5
0.6
0.7
0.8
data sparsity

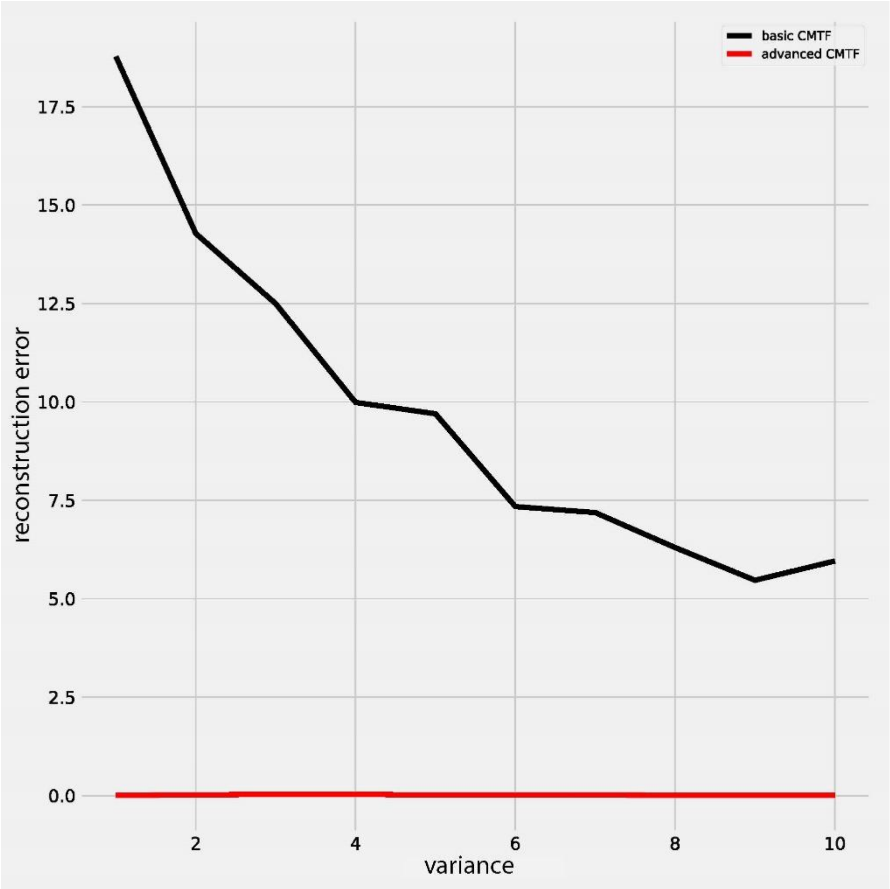

Figure S1. Simulation study on the effect of different data variance and data sparisity on factorization. A: Simulated factor matrices were randomly drawn from normal distribution $\boldsymbol{N(10, sigma)}$, where sigma ranging from 1 to 10, these factor matrices were used to build simulated tensor and matrix respectively, applying identical weights on tensor and matrix. The error was evaluated on basic CMTF and advanced CMTF.B: Simulated factor matrices were randomly drawn from normal distribution $\boldsymbol{N(10, 1)}$, simulated tensor and matrix were built using the same way as above. Then a proportion ranging from 0 to 0.8 of data was randomly masked from simulated tensor and matrix to simulate different data sparsity. The error was evaluated on basic CMTF and advanced CMTF.

Table S1. Description of datasets used for validating the CMTF framework. The table summarizes key characteristics of each dataset, including the number of detected microbial taxa, annotated functional pathways, and metabolites, along with the total number of samples analyzed. Tensor sparsity refers to the proportion of missing or zero entries in the tensor representation of the data, while matrix sparsity refers to the proportion of missing or zero entries in the metabolomic data matrix.

	Number of Detected Microbes	Number of Annotated Pathways	Number of Detected Metabolites	Number of Samples	Tensor Sparsity	Matrix Sparsity
Flight Cabin	620	468	1335	27	0.987	0
IBDMDB	450	428	596	106	0.974	0
COVID-19 gut related	476	337	284	49	0.978	0

Table S2. IBD-related microbial biomarkers.

IBD-related Microbial Biomarkers		Source
Significant different abundance between dysbiosis and non-dysbiosis groups	*Alistipes*	IBDMDB analysis detected
	F. prausnitzii	*Experimentally validated*
	Subdoligranulum	*Experimentally validated*
	R. torques	*Experimentally validated*
	R. gnavus	*Experimentally validated*
	P.copri	*Experimentally validated*
Greatest longitudinal shifts	*R.intestinalis*	IBDMDB analysis detected
	F.prausnitzii	*Experimentally validated*
	Bacteroides fragilis	IBDMDB analysis detected
	Bacteroides vulgatus	IBDMDB analysis detected
	Bacteroides ovatus	IBDMDB analysis detected
	Bacteroides uniformis	IBDMDB analysis detected
	E. rectale	IBDMDB analysis detected
	E.coli	IBDMDB analysis detected

Table S3. IBD-related metabolic biomarkers.

IBD-related Metabolic Biomarkers		Source
Significant different abundance between dysbiosis and non-dysbiosis groups	Nicotinuric acid	Experimentally validated
	Adrenate	Experimentally validated
	Hydroxycotinine	IBDMDB analysis detected
	Uridine	IBDMDB analysis detected
	Nicotinate	Experimentally validated
	Butyrate	Experimentally validated
	Propionate	Experimentally validated
	Cholate	Experimentally validated
	Taurocholate	Experimentally validated
	Glycocholate	Experimentally validated
	Lithocholate	Experimentally validated
	Acylcarnitine	IBDMDB analysis detected
Greatest longitudinal shifts	Urobilin	IBDMDB analysis detected
	Urate	IBDMDB analysis detected
	HILn_QI1594	IBDMDB analysis detected
Predicted interaction with microbes	NH4_C56:1 TAG	MMvec detected
	NH4_C48:4 TAG	MMvec detected
	NH4_C48:3 TAG	MMvec detected
	NH4_C44:1 TAG	MMvec detected
	NH4_C44:0 TAG	MMvec detected
	NH4_C56:4 TAG	MMvec detected
	NH4_C52:4 TAG	MMvec detected
	NH4_C54:6 TAG	MMvec detected
	NH4_C54:5 TAG	MMvec detected
	NH4_C36:3 DAG	MMvec detected
	NH4_C51:3 TAG	MMvec detected
	NH4_C52:1 TAG	MMvec detected
	NH4_C56:6 TAG	MMvec detected
	C51:1 TAG	MMvec detected
	NH4_C16:1 MAG	MMvec detected
	NH4_C38:5 DAG	MMvec detected
	NH4_C32:2 DAG	MMvec detected
	C32:2 DAG	MMvec detected
	NH4_C34:2 DAG	MMvec detected
	NH4_C22:5 CE	MMvec detected
	NH4_C34:3 DAG	MMvec detected
	NH4_C20:5 CE	MMvec detected
	NH4_C36:4 DAG	MMvec detected
	C34:3 DAG	MMvec detected
	NH4_C20:4 CE	MMvec detected
	C34:2 DAG	MMvec detected
	NH4_C18:3 CE	MMvec detected
	C38:5 DAG	MMvec detected
